# The European Commission’s Task Force on Bioterrorism

**DOI:** 10.3201/eid0910.030368

**Published:** 2003-10

**Authors:** Anders Tegnell, Philippe Bossi, Agoritsa Baka, Frank Van Loock, Jan Hendriks, Solvejg Wallyn, Georgios Gouvras

**Affiliations:** * Task Force on Biological and Chemical Agent Threats, European Commission, Luxembourg, Belgium

## Abstract

In response to the increased threat of bioterrorism, a task force on health security was established in the European Commission. Task force members address a broad range of issues related to preparedness for and response to bioterrorist events and seek to bring about a greater collaboration between the European Union member states.

The deliberate release of anthrax in the United States of America in September and October 2001 completely changed the international perception of the risk of bioterrorism. Before that time, public health preparedness for bioterrorism was not a political priority in the European Union (EU) member states. Preparedness and response plans and actions have since been given a higher priority on EU member agendas. Bioterrorism also initiated a discussion on the need to improve preparedness through reinforcing existing public health structures that are in charge of monitoring and controlling diseases.

On October 19, 2001, at the European Council in Ghent, Belgium, the Council of Ministers of the EU and the European Commission (EC) were asked by the heads of state and government to prepare a program to improve the cooperation between member states for the evaluation of risks, alerts, and intervention, the storage of the necessary means, and the collaboration in the field of research.

In parallel with these developments at the European level, many well-focused national activities were also carried out. Countries reexamined their preparedness plans and adapted them to the new threats, and legislation needed to implement countermeasures was reviewed. The national authorities also discussed the necessity of stockpiling medicines and other medical supplies and most upgraded their stocks of vaccines and antibiotics, especially smallpox vaccine. To improve the ability to recognize a deliberate release, countries developed training programs for different kinds of first responders.

On the initiative of the European Commissioner for Health and Consumer Protection, the Health Security Committee (HSC) was set up with representatives of the ministries of health from each member state. Following a proposal by the commission, the HSC agreed on December 17, 2001, on a program for cooperation on preparedness and response to biologic and chemical agent attacks (or Health Security Programme). The program comprises 25 actions grouped under four objectives (available from: URL: http://europa.eu.int/comm/health/ph_threats/Bioterrorisme/bioterrorisme_en.htm). The overall goal is to improve cooperation between the member states with the aid of the commission and to facilitate collaboration between the different national authorities involved in public health preparedness for bioterrorism. During this process, gaps in resources and methods are identified and projects initiated to fill these gaps. As is standard practice in many sectors of activity at EU level, existing examples of preparedness in the member states are shared and can be extended or adapted for use throughout the EU. Projects and activities under the health security program are funded through existing funds, especially from the budget foreseen for the EU’s public health programs. Other funds such as from the sixth framework program for research could also be used for activities of relevance to the health security program.

Since the beginning of May 2002, a 15-member strong task force has been set up by the Directorate-General of Health and Consumer Protection, comprising nine national experts seconded from different institutions in the EU countries and six commission officials. The task force includes representatives from the Robert Koch Institute and the Paul Erhlich Institute (Germany), the Scientific Institute of Public Health (Belgium), the Swedish Institute for Infectious Diseases Control (SMI, Sweden), the National Institute of Public Health and the Environment (RIVM, Netherlands), the Hellenic Institute for Infectious Disease Control (Greece), the Italian National Institute for Health (Italy), as well as the Pitié-Salpêtrière Hospital in Paris (France). The task force’s main objective is to implement the health security program. To this end, members have initiated further activities with key players in the member states, some of which are described below.

One of the initial priority activities was the setting up of a mechanism for rapid information exchange, consultation, and coordination on all aspects dealing with bioterrorism. All designated authorities of the EU member states have now been connected through this network by means of email, fax, and telephone. This tool makes it possible for the task force and the nine member states to disseminate information rapidly about suspected or confirmed incidents and for national authorities to evaluate measures planned with their implications for EU and for other member states. The task force is available 24 hours a day, 7 days a week to facilitate the process. By May 2003, the system had been used in two major EU-wide exercises and for nine alerts about suspicious events communicated by the member states.

The early detection of a deliberate release or bioterrorist attack is an essential part of a program of preparedness. One essential tool to make this possible will be the regular surveillance activities already in place at national and European level. Certain modifications to these systems, such as the addition of certain diseases considered as having a high potential for deliberate release, would have to be done to make the systems better adapted to the new challenges of bioterrorism. To achieve this, the Community Network on Communicable Diseases (the already existing mechanism for EU collaboration in the areas of infectious disease surveillance and control) extended its list of diseases to be reported, to include additional threats such as tularemia, anthrax, Q-fever, and smallpox.

When the task force was established, several lists of agents that could be involved in a biologic attack and considered of major bioterrorist threat had been established and published. Against this background, an approach more suitable to the demands for focused action in Europe was developed though a matrix model designed to provide an evaluation of the public health impact of any given agent that could be used in bioterrorism. The matrix model may also be used to identify areas where the public health response needed strengthening for any given agent. This means that for a given type of action a different set of agents would emerge as deserving the highest attention. The matrix model is currently being validated and has produced lists targeting specific areas of public health preparedness, which are currently being reviewed by members of the HSC. Using this model, identifying the public health impact of new threats should be possible, such as severe acute respiratory syndrome (SARS) (once it has been better characterized), multidrug-resistant tuberculosis, or any other new or drastically changed disease-causing agent.

The clinical management, including recognition, of many of the diseases caused by the bioterrorist agents is likely to be difficult, since they are, to a large extent, unknown to the average clinician in Europe today. To provide clinicians with guidance and a source of generally agreed-upon information, 10 different articles on 48 major agents have been compiled (*Bacillus anthracis* (anthrax), *Yersinia pestis* (plague), *Francisella tularensis* (tularemia), smallpox and monkeypox viruses, *Clostridium botulinum* (botulism), hemorrhagic fever viruses, encephalitis viruses, *Coxiella burnetii* (Q fever), *Brucella* species (brucellosis), *Pseudomonas mallei* (glanders), and *Pseudomonas pseudomallei*). They have been sent for comments to experts in all member states, have been adopted by HSC, and will be made available to the European medical community. Their publication in a peer-reviewed medical journal is currently being considered.

The laboratory capacity required to diagnose most of the potentially toxic and infectious agents that might be used in case of deliberate release is believed to exist within the EU as a whole; however, no member state has the complete spectrum of the diagnostic capabilities needed. To improve the collective preparedness and to make more efficient use of the available resources, the laboratory capacities already in place are currently under review. As a long-term goal these capabilities would be made available to all member states of the EU. Additionally, more concrete collaboration between laboratories was initiated to share techniques and participate in common quality assurance schemes. Assisted by the task force, the P4 (U.S. BSL-4) laboratories in the EU are currently forming a network that will enhance their research activities through common projects. They have been asked to develop procedures to make P4 diagnostics available to all member states and to handle, under high safety level requirements, a substantial workload of analysis of environmental samples that might arise following a series of bioterrorist incidents or threats.

The European Agency for the Evaluation of Medicinal Products (EMEA) has already developed guidelines on the use of authorized pharmaceuticals in case of deliberate release of biologic and chemical agents (available from: URL: http://www.emea.eu.int/index/indexh1.htm). This includes guidelines for treatment and prophylaxis of the agents on the so-called CDC A-list of the most important threats as bioterrorist agents (*1*).

Stockpiling of medicines and vaccines (in particular, smallpox vaccines) has been extensively discussed between the different member states and the EC. Although the member states initially looked into the possibility of an EU-level vaccine stockpile, the member states opted to establish national stockpiles sufficient for their projected needs.

Smallpox has become the model for much of the work performed in the area of preparedness for bioterrorist incidents. Vaccination is certainly an essential countermeasure against possible smallpox threats, yet finding the right balance under different vaccine administration scenarios between personal and societal cost-benefit ratios has proved extremely difficult. To find optimal solutions, various attempts have been made to construct appropriate mathematical models. Currently a group of European modelers has been called by the task force to work together in a network to develop models for EU purposes. In addition, this group has been asked to give expert opinions on new models published and to establish an infrastructure for data-exchange that could be used for real-time modeling, should the need arise.

Many member states have developed (or are in the process of developing) specific preparedness plans for smallpox. The task force has put in place a compilation and review process, which includes the tabulation and comparison of these plans to improve the understanding of how and with what measures individual countries respond to specific triggers and events and how components of their plans, which are important for the whole EU, will interact.

National authorities had already developed substantial capacities to deal with chemical incidents. Much of the EU work on chemical incidents has been done in close collaboration with other organizations like World Health Organization (WHO) and Organization for the Prohibition of Chemical Weapons. A special working-group has been formed under the auspices of HSC, and a project will be initiated to take work on certain key aspects forward.

In most areas investigated, existing European expertise was identified that could ensure a high level of preparedness against biologic or chemical agents. Considerable effort is needed, however, to determine the exact kind of expertise needed for specific cases and to identify specialists and develop the modalities required for effective sharing of this expertise between countries. As a first step, a plan for the development of a common directory of experts has been developed, and countries have been asked to make their national expertise available on behalf of the EU. Further modalities will be developed when this first step is implemented.

Better preparedness should also include improved coordination between the commission and international organizations working in this area such as the WHO and the parties to the Global Health Security Action Initiative (Canada, France, Germany, Italy, Japan, Mexico, the United States, the United Kingdom, and the European Commission with WHO participating as a resource). Multiple initiatives regarding coordination of actions in this area are being pursued.

The EU program on health security is expected to be concluded in November 2003. However, this deadline likely does not provide enough time to complete the activities anticipated in many of the areas included in the 25-point action program. An extension of the task force’s duration is therefore being sought. In the areas where activities have taken place, a centrally launched initiative at EU level has been found to be beneficial both for the member states and for the EU as a whole. The work has opened new communication channels between member states, improving both the understanding of the preparedness in other countries as well as giving the possibility to learn from good practice and tried solutions. The EC has also gained experience in working with a group of experts who initiate activities with more operational effectiveness than has been the case in the past. This experience will be very useful in the continued discussion on the development of a European Centre for Disease Prevention and Control since the task force can be seen as a precursor to this centre, albeit in a narrow area of activity. During the remaining 6 months and the period of extension, if it is approved, the work in the area of bioterrorism will continue to strengthen the capabilities at EU level to respond to biologic and chemical threats. The capabilities to manage diseases in emergency situations in general will be improved, and knowledge gained will be most useful for other areas of emergency preparedness.
